# LINC00992 contributes to the oncogenic phenotypes in prostate cancer via targeting miR-3935 and augmenting GOLM1 expression

**DOI:** 10.1186/s12885-020-07141-4

**Published:** 2020-08-11

**Authors:** Jianheng Chen, Xiaodong Liu, Kunbin Ke, Jianan Zou, Zhan Gao, Tomonori Habuchi, Xuezhen Yang

**Affiliations:** 1 Department of Urology, the First Affiliated Hospital of Kunming Medical University, Kunming, 650032 Yunnan China; 2 Department of Urology, the First Affiliated Hospital of Anhui University of Chinese Medicine, Hefei, 230031 Anhui China; 3 Department of Urology, Xiyuan Hospital, China Academy of Chinese Medical Sciences, Beijing, 100091 China; 4 Department of Urology, Akita University School of Medicine, Akita, 010-8543 Japan; 5 Department of Urology, the Second Affiliated Hospital of Bengbu Medical College, 220 Hongye Road, Bengbu, 233000 Anhui China

**Keywords:** INC00992, miR-3935, GOLM1, Prostate cancer

## Abstract

**Background:**

Accumulating evidence has revealed the critical role of long non-coding RNAs (lncRNAs) in cellular processes during tumor progression. As documented in cancer-related literatures, LINC00992 expression is associated with cancer progression, whereas its function in tumors including prostate cancer has not been characterized yet.

**Methods:**

Data from GEPIA database suggested LINC00992 expression in prostate cancer tissues. The expression levels of RNAs were monitored via qRT-PCR. Western blot evaluated the levels of proteins. The proliferation, apoptosis and migration of prostate cancer cells were assessed by CCK-8, EdU, TUNEL, Transwell and wound healing assays. Luciferase reporter, RNA pull down and RIP assays were applied to detect the interplays among LINC00992, miR-3935 and GOLM1.

**Results:**

Elevated levels of LINC00992 and GOLM1 were detected in prostate cancer tissues and cells. LINC00992 exerted facilitating functions in prostate cancer cell proliferation and migration. Mechanically, LINC00992 interacted with and negatively regulated miR-3935 to elevate GOLM1 expression in prostate cancer cells. In addition, the in vitro suppressive effect of silenced LINC00992 on prostate cancer cell proliferation and migration was reversed by GOLM1 upregulation. Likewise, LINC00992 depletion restrained tumor growth in vivo was offset by enhanced GOLM1 expression.

**Conclusions:**

LINC00992 competitively bound with miR-3935 to elevate GOLM1 expression and therefore facilitate the oncogenic phenotypes of prostate cancer cells, implying a potential LINC00992-targeted therapy for prostate cancer.

## Background

Clinically, prostate cancer manifests as a dominating cause of male-related death worldwide and is characterized as the most continually occurred tumor among men in the United States [1]. The biggest challenge is the detectable bone metastases in roughly 80% advanced prostate cancer [2]. Virtually all prostate cancer patients during 2 years’ androgen deprivation treatment inevitably undergo castration-resistance, which contributes to the poor clinical consequences in prostate cancer [3]. However, the mechanism underlaid prostate cancer remains mostly unknown.

The widely studied long noncoding RNAs (lncRNAs) are transcribed from non-protein-coding human genome and have more than 200 nt in length [4]. LncRNAs are increasingly functionally identified and experimentally consolidated to be related to tumor neoplasia and progression in diverse cancers [5]. Additionally, lncRNAs with dysregulation can functionally modulate tumor development from multiple pathological aspects such as cell proliferation, drug-resistance, and metastasis [6–8]. For example, lncRNA A1BG-AS1 inhibits cell proliferation and invasion in hepatocellular carcinoma via targeting miR-216a-5p [9]. LncRNA LOC730100 sponges miR-760 from FOXA1 to accelerate cell proliferation and invasion in glioma [10]. LncRNA SNHG16 functions as an oncogene in hepatocellular carcinoma [11]. Long intergenic non-protein coding RNA 992 (LINC00992) is a novel lncRNA that has been previously revealed to be elevated in tumors and substantiated as a master regulator for chemo-resistance [12]. Besides, LINC00992 has been uncovered as an elevated lncRNA in prostate cancer [13], which is consistent with the detection from GEPIA database. Despite that, no previous study has given a comprehensive explanation about the precise function or detailed mechanism of LINC00992 in prostate cancer.

In past decades, the fact that lncRNAs function in tumors depending on their secondary or tertiary structures has been reported in many cancer-linked studies. For instance, in the nucleus, lncRNAs are entitled to work as molecular scaffolds or alternative splicing assistants [14, 15]. On the contrary, lncRNAs dispersing in cytoplasm influence downstream mRNA translation or degradation through serving as miRNA sponges [16, 17]. For example, TNF-α-induced lncRNA LOC105374902 promotes the malignant behaviors of cervical cancer cells by acting as a sponge of miR-1285-3p [18]. LncRNA TTN-AS1 promotes papillary thyroid cancer tumorigenesis by regulating miR-153-3p/ZNRF2 axis [19]. Nevertheless, whether LINC00992 could exert its functions in prostate cancer via its sponging role of certain miRNA remains unknown.

We conducted this research aiming to explore the function or probable mechanism of LINC00992 in prostate tumor, which might enrich the understanding in terms of prostate tumor pathology and contribute to a wider applied scope.

## Methods

### Tissue samples

The prostate cancer tissue samples and matched peri-tumor tissue samples were collected from 60 patients diagnosed with prostate cancer, under the approval of the Ethics Committee of the First Affiliated Hospital of Kunming Medical University. Each participant did not receive radiotherapy and chemotherapy prior to tissue collection, and signed the written informed consents before this study. All samples were snap-frozen in liquid nitrogen and then stored at -80 °C until required for further analysis.

### Cell culture

The prostate epithelial cell line (RWPE-1; CRL-11609) and prostate cancer cells, including PC3 (CRL-1435), LNCaP (CRL-1740), C4–2 (CRL-3314), and DU145 (HTB-81), were all purchased from American Type Culture Collection (ATCC; Manassas, VA, USA) in October 2017. All cells were cultured as recommended in Dulbecco’s modified Eagle’s medium containing 10% FBS (GIBCO, MA, USA) under the condition of a cell incubator with 5% CO_2_ at 37 °C. Before using in this study, all cell lines were authenticated by STR profiling and tested for mycoplasma contamination in June 2018.

### Cell transfection

LINC00992 shRNA or negative control shRNA, and pcDNA3.1/LINC00992, pcDNA3.1/GOLM1 or its empty control pcDNA3.1 plasmid were chemically synthesized and provided by Gene Pharma (Shanghai, China). MiR-3935 mimics, miR-3935 inhibitor and their related negative controls (NC-mimics, NC-inhibitor) were all purchased for upregulating or downregulating miR-3935 from Ribobio (Guangzhou, China). In line with the directions of LipofectamineTM RNAiMAX Transfection Reagent (Thermo Fisher Scientific), transfection of these plasmids into DU145, PC3 and RWPE-1 cells was conducted and qRT-PCR checked the transfection efficiency. The sequences were as follows: sh-NC: 5′-CCGGTAGTAATTGACAACCATTATACTCGAGTATAATGGTTGTCAATTACTATTTTTG-3′, sh-LINC00992#1: 5′-CCGGATTATCCAAGAGTATTAACATCTCGAGATGTTAATACTCTTGGATAATTTTTTG-3′, sh-LINC00992#2: 5′-CCGGTGTTAGATGATCATTGAGGTGCTCGAGCACCTCAATGATCATCTAACATTTTTG-3′, sh-LINC00992#3: 5′-CCGGTTACCTAATCAGTAGATGCAGCTCGAGCTGCATCTACTGATTAGGTAATTTTTG-3′; NC-mimics: 5′-UCAGGUAGGGCUCAAACCAACC-3′, miR-3935 mimics: 5′-UGUAGAUACGAGCACCAGCCAC-3′; NC-inhibitor: 5′-CUGGCUUUAGGGUGCCACUUAG-3′, miR-3935 inhibitor: 5′-GUGGCUGGUGCUCGUAUCUACA-3′.

### Quantitative real-time PCR (qRT-PCR)

On the basis of the instructions of Trizol reagent (Invitrogen, USA), RNA extraction was executed in prostate cancer cells. After the examination of RNA purity with spectrophotometry, cDNA was obtained from above RNA with reverse transcription kit (4,368,814, Thermo Fisher Scientific, shanghai, China). qRT-PCR analysis was devised with the aid of a BioRad CFX96 system and SYBR green was applied for investigating the RNA levels. The internal reference for LINC00992 and mRNAs was GAPDH whereas that for miRNAs expression was U6. Relative expression was assessed based on the method of 2^−ΔΔCt^.

### Western blot

Protein content in cells was determined by western blot analysis. RIPA lysis buffer (Beyotime, Shanghai, China) was adopted for cell lysing, followed by the evaluation of the protein concentration with BCA Protein Assay Kit (P0011, Beyotime Tech.). 10% SDS-PAGE gel was applied for separating proteins (20 μg protein per sample) and then proteins were transferred onto 0.2 μm PVDF membranes (Bio-Rad, Hercules, CA, USA). Antibodies including anti-GOLM1 (ab109628, 1/1000; Abcam, Cambridge, UK), anti-PCNA (ab92552, 1/1000; Abcam), anti-CDK2 (ab32147, 1/1000; Abcam), anti-Cyclin D1 (ab40754, 1/1000; Abcam), anti-Bax (ab32503, 1/1000; Abcam), anti-Bcl-2 (ab32124, 1/1000; Abcam), anti-MMP2 (ab97779, 1/1000; Abcam), anti-MMP9 (ab38898, 1/1000; Abcam), anti-p-Src (ab40660, 1/1000; Abcam), anti-Src (ab47405, 1/1000; Abcam), anti-p-FAK (ab81298, 1/1000; Abcam), anti-FAK (ab131435, 1/1000; Abcam), anti-GAPDH (ab8245, 1/1000; Abcam) and anti-Tubulin (ab7291, 1/10000, Abcam), were applied to probe the membranes overnight at 4 °C. After that, the membranes were further incubated for 1 h with HRP-conjugated secondary antibody (Santa Cruz, Co. Ltd., Sant Cruz, CA, USA) at room temperature. ECL Substrates (Millipore, Billerica, MA, USA) was utilized for the visualization of signals, followed by exposure to X-film (Kodak, Rochester, NY, USA). The quantification of immunoblots was conducted with the aid of imageJ software (National Institute of Health, Bethesda, MD, USA), with GAPDH or Tubulin as the normalizer as needed.

### Luciferase reporter assay

Fragments of full-length LINC00992 with wild-type or mutant binding sites for miR-3935 and sequences of GOLM1 3’UTR containing wild-type or mutated miR-3935 binding sites were inserted into the pmirGLO vectors (Promega, Madison, WI, USA) for the construction of reporters (LINC00992-WT, LINC00992-MUT, GOLM1-WT, GOLM1-MUT). Then, the four reporters and miR-3935 mimics or miR-3935 inhibitor (GenePharma) were co-transfected into DU145 and PC3 cells applying lipofectamine2000 (Invitrogen), as needed. Forty-eight hours later, Dual-Luciferase Reporter Assay System (Promega) was employed for the examination of the luciferase activity. GloMax® Discover Multimode Microplate Reader (Promega) assessed the ratio of Firefly/Renilla luciferase activity and the activity of Renilla was the normalized control.

### RNA immunoprecipitation (RIP) assay

According to the direction for usage of Magna RIP™ RNA Binding Protein Immunoprecipitation Kit (17–700, Millipore), RIP assay was strictly performed. RIP lysis buffer was firstly applied to treat the transfected DU145 and PC3 cells. Afterwards, the obtained cell lysates were processed with magnetic beads integrated with human anti-Ago2 antibodies (ab32381; Abcam, MA, USA) or anti-IgG (AP162-KC, Millipore). Following the recovery of antibody by the protein A/G beads, qRT-PCR detected the levels of LINC00992, miR-3935, and GOLM1 mRNA in the precipitates. IgG worked as the negative control for the normalization of RNA-IPs.

### RNA isolation of nuclear and cytoplasmic fractions

The dispersion of LINC00992 in the prostate cancer cells was assayed as described previously [20]. The isolation of cytosolic and nuclear sections was executed following the protocol of PARIS™ Kit (AM1921, Invitrogen). RNA levels of U1 (nuclear control), GAPDH (cytoplasmic control) and LINC00992, were all estimated by qRT-PCR analysis.

### Fluorescence in situ hybridization (FISH) assay

In line with the recommendation of Ribo™ FISH Kit (C10910, Ribobio, Guangzhou, China), FISH analysis was implemented for testing the presence of LINC00992 in prostate tumor cells. Ribobio Company synthesized the LINC00992 probes, labeled by Cy3 fluorescent dye. Following the fixation by 4% paraformaldehyde and 0.5% Triton X-100 permeabilization, DU145 and PC3 cells were subsequently blocked in pre-hybridization buffer/blocking solution. Then incubation of cells with probe/hybridization buffer was later performed. Next day, after rinsing and Hoechst staining, the fluorescence was measured under a confocal laser scanning microscope (Zeiss, Germany).

### Cell counting kit-8 (CCK-8) assay

For the viability assessment in DU145, PC3 and RWPE-1 cells, CCK-8 assay was implemented as described previously [21]. Cell viability was monitored at 24, 48, 72 and 96 h. In short, after being seeded onto 96-well plates and cultured for indicated times, cells were processed with 10 μl of CCK-8 solution. Then, a microplate reader examined the absorbance values at the wavelength of 450 nm.

### 5′-ethynyl-2′-deoxyuridine (EdU) incorporation assay

Cell proliferation was examined through EdU assay as described previously [22], by using Click-iT EdU Alexa Fluor 488 Imaging Kit (C10086, Invitrogen). After 48 h of transfection, EdU staining was carried out as instructed. The observation and calculation of EdU-positive cells was proceeded under the fluorescence microscopy.

### Transferase-mediated dUTP nick end labeling (TUNEL) staining

TUNEL assay was carried out as described previously [23], for probing DU145 and PC3 cell apoptosis with the assistance of an In Situ Cell Death Detection Kit (11,684,795,910, Roche, Mannheim, Germany). TUNEL-positive cells were recorded under a light microscope (× 400) from 10 visual fields which were chosen at random.

### Transwell migration assay

The application of transwell chambers with pore size 8-μm (Corning Costar, Cambridge, MA, USA) was aimed for detecting cell migration in strict line with the instructions. Cells that were previously suspended in serum-free RPMI-1640 media were seeded into the upper chamber. RPMI-1640 medium containing 20% FBS was supplemented in lower chamber as a chemoattractant. Cells in the filters following 48 h incubation were immobilized in methanol and went through 0.1% crystal violet staining. The images of cells migrated through the filters were obtained and counted under the microscope.

### Wound healing assay

The DU145, PC3 and RWPE-1 cells (2 × 10^3^ cells/well) were prepared on glass culture dishes and cultivated at 37 °C for a whole night to allow cells adhered to the plates, followed by the straight scratch made with a plastic pipette tip after cell samples reached 80% confluence. Later, cells were rinsed in PBS to clear the detached cells. Finally, the wounds at 0 and 24 h were imaged via a light microscopy (Olympus, Tokyo, Japan).

### In vivo experiment

Sixteen six-week-old male BALB/C athymic nude mice were commercially available from the National Laboratory Animal Center (Beijing, China) and maintained in SPF-grade animal lab. All animal-related protocols were approved by the Animal Research Ethics Committee of the First Affiliated Hospital of Kunming Medical University. The in vivo experiment was undertaken via subcutaneous injection of 5 × 10^6^ DU145 cells into the nude mice, while the DU145 cells injected into indicated four groups of mice were transfected with sh-NC, sh-LINC00992#1, sh-LINC00992#1 + pcDNA3.1, or sh-LINC00992#1 + pcDNA3.1/GOLM1. Tumor volume was monitored every 4 days. 28-day after injection, nude mice were sacrificed via cervical dislocation and then tumor samples were carefully dissected for weight assessment and hematoxylin and eosin (H&E) staining.

### Immunohistochemistry (IHC)

The tumor samples collected from in vivo experiments were treated with 4% PFA, dehydrated and embedded in paraffin. Afterwards, the paraffin-embedded sections (4 μm) were prepared for IHC assay as described previously [24], by use of the anti-Ki67 and anti-PCNA antibodies (Abcam).

### Statistical analysis

SPSS 17.0 statistical software (SPSS, Armonk, NY, USA) was employed in the processing of data from three biological replicates, and data were expressed as mean ± SD. Significance of difference within two groups was determined using Student’s t-test, while that among no less than two groups was tested via one-way or two-way ANOVA. *P* < 0.05 was considered as the threshold of significance.

## Results

### LINC00992 is overexpressed in prostate cancer and regulates cell proliferation, apoptosis and migration

**Fig. 1:**
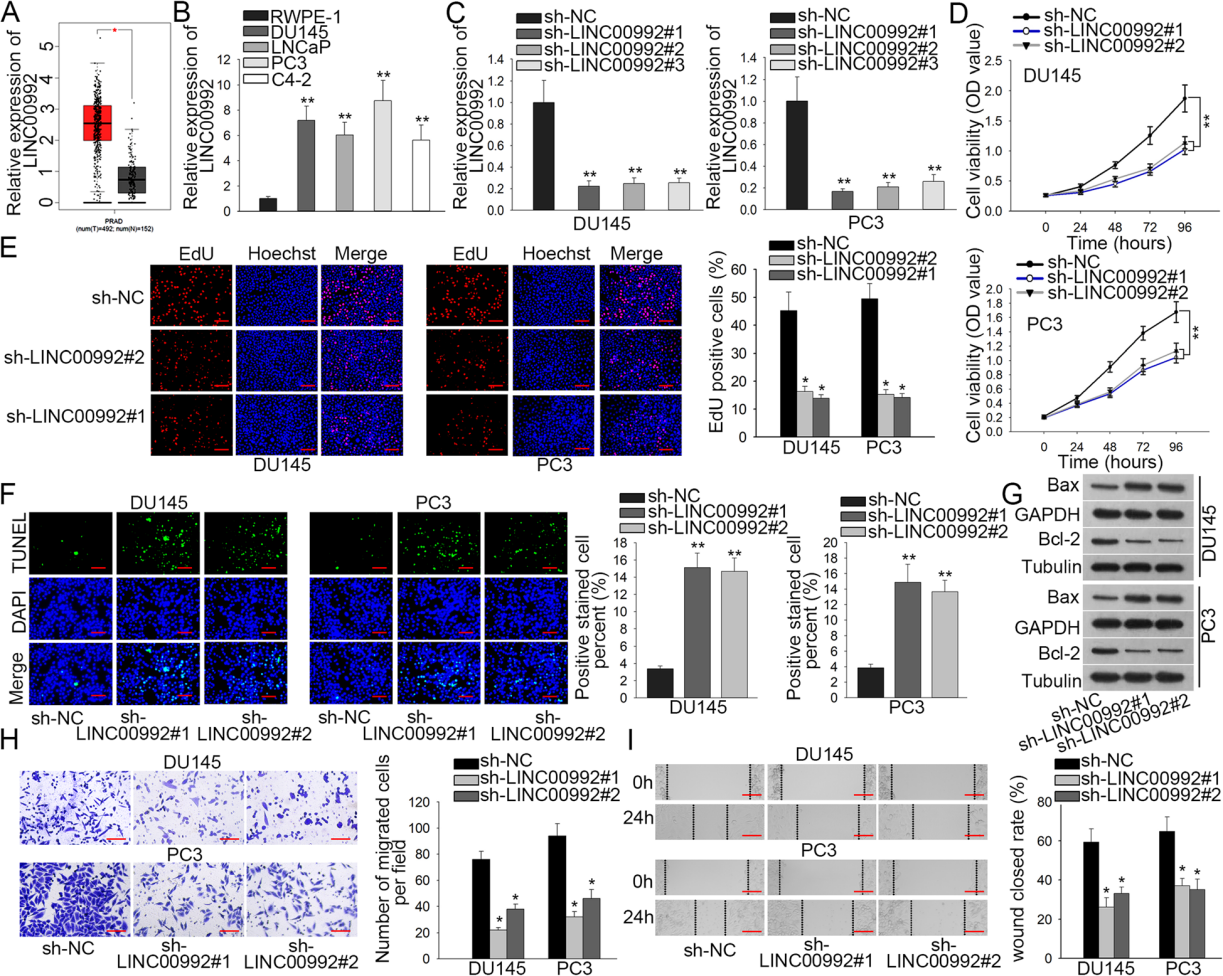
LINC00992 was overexpressed in prostate cancer and regulates cell proliferation, apoptosis and migration. **a** GEPIA database demonstrated the overexpression of LINC00992 in tumor tissues in contrast to adjacent normal ones. **b** LINC00992 expression was detected by qRT-PCR in four prostate cancer cell lines and control RWPE-1 cells. **c** LINC00992 expression was monitored by qRT-PCR in DU145 and PC3 cells after transfection with shRNAs targeting LINC00992. sh-NC was used as the negative control. **d** The viability of DU145 and PC3 cells was estimated through CCK-8 assay following LINC00992 depletion. **e** The proliferation of DU145 and PC3 cells was investigated after LINC00992 depletion via EdU assay. Scale bar = 200 μm. **f** The apoptosis of DU145 and PC3 cells transfected with sh-LINC00992#1/2 or sh-NC was estimated via TUNEL assay. Scale bar = 200 μm. **g** Western blot analysis was applied to examine the expression of apoptosis-related proteins. **h-i** The migration of DU145 and PC3 cells was analyzed via Transwell migration assay (scale bar = 100 μm) and wound healing assay (scale bar = 100 μm) after inhibiting LINC00992 expression. The full-length images for blots in Fig. 1g were presented in Supplementary figure 4. *P < 0.05, **p < 0.01

LINC00992 expression pattern in prostate cancer was acquired from online GEPIA database. As a result, LINC00992 was considerably upregulated in PRAD (prostate adenocarcinoma) tissues relative to normal ones (Fig. 1a). After detecting LINC00992 expression in tissue samples obtained from patients with prostate cancer, we observed that LINC00992 expression was higher in prostate cancer tissues than that in peri-tumor tissues (Figure S1A). Moreover, clinical data showed that higher expression of LINC00992 in prostate cancer patients was associated with lower survival rate (Figure S1B). Furthermore, LINC00992 expression in the prostate cancer cells and RWPE-1 cells was evaluated by qRT-PCR. Consequently, higher level of LINC00992 was exhibited in prostate cancer cells than that in RWPE-1 cells (Fig. 1b), which was completely consistent with the result presented in previous discovery [13]. Particularly, DU145 and PC3 cells expressed the highest level of LINC00992 and was thereby chosen for the later assays. For silencing LINC00992, special shRNAs targeting LINC00992 was transfected into DU145 and PC3 cells and the efficiency was corroborated in qRT-PCR (Fig. 1c). And then the data from CCK-8 assay revealed that LINC00992 depletion suppressed the proliferation of DU145 and PC3 cells (Fig. 1d). As expected, a declined proportion of EdU positive cells was observed after knocking down LINC00992 (Fig. 1e), suggesting the suppressive effect of LINC00992 deficiency on prostate cancer cell proliferation. Additionally, the expression levels of proliferation-related proteins (PCNA, CDK2 and Cyclin D1) were all reduced by silenced LINC00992 (Figure S1C). On the contrary, TUNEL assay uncovered that LINC00992 knockdown facilitated cell apoptosis (Fig. 1f). Meanwhile, western blot analysis revealed that LINC00992 knockdown promoted the apoptosis of DU145 and PC3 cells, as Bax protein level was increased whereas Bcl-2 protein level was decreased after LINC00992 was silenced in these two cells (Figs. 1g, Figure S1D). Further, Transwell and wound healing assays indicated that the migration of DU145 and PC3 cells was retarded by LINC00992 depletion (Fig. 1h-i). Likewise, the expression of migration-related molecular markers (MMP2, MMP9, p-Src and p-FAK) was decreased by LINC00992 inhibition (Figure S1E). To further verify the biological role of LINC00992 in prostate cancer, we carried out gain-of-function assays in RWPE-1 cells. After overexpressing LINC00992 in RWPE-1 cells (Figure S2A), cell proliferation was promoted (Figure S2B-C). As expected, the expression of PCNA, CDK2 and Cyclin D1 was decreased by upregulation of LINC00992 (Figure S2D). Similarly, LINC00992 upregulation facilitated cell migration (Figure S2E-F). In addition, upregulating LINC00992 resulted in the elevated protein levels of MMP2, MMP9, p-Src and p-FAK (Figure S2G). All these data elucidated that LINC00992 could facilitate cell proliferation and migration whereas suppress cell apoptosis in prostate cancer.

### MiR-3935 is targeted by LINC00992

**Fig. 2:**
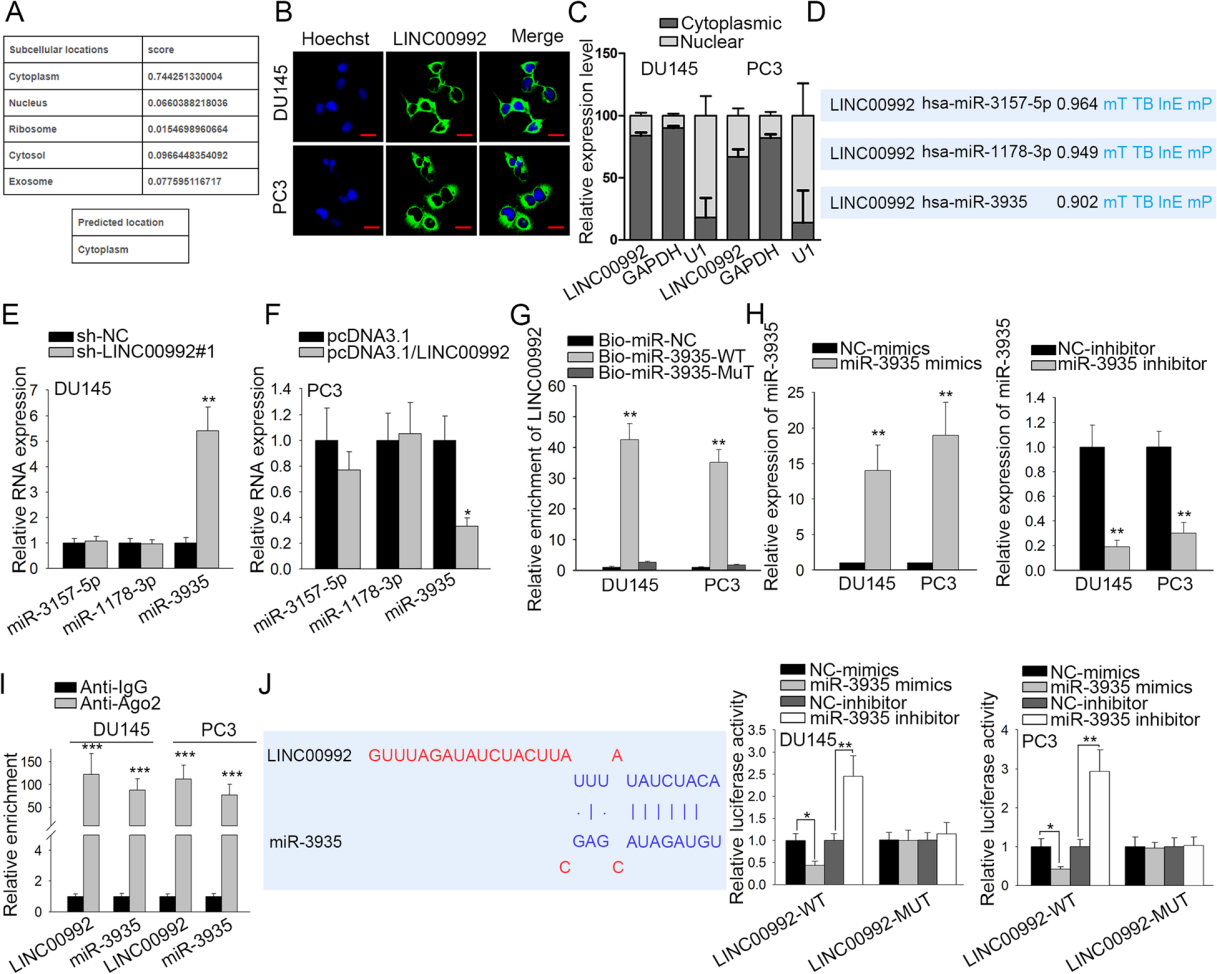
MiR-3935 was targeted by LINC00992. **a** LncLocator predicted LINC00992 subcellular location. **b** FISH analysis of LINC00992 distribution in prostate cancer cells. Scale bar = 30 μm. **c** RNA isolation of nuclear and cytoplasmic fractions assayed the subcellular distribution of LIN00992 in prostate cancer cells. **d** Top three miRNAs which might interact with LINC00992 were predicted by DIANA-lncBase. **e** After transfection of LINC00992-silencing plasmids, the expression of miR-3157-5p, miR-1178-3p and miR-3935 was examined via qRT-PCR. **f** Following LINC00992 upregulation, qRT-PCR tested the levels of miR-3157-5p, miR-1178-3p and miR-3935 in DU145 and PC3 cells. **g** RNA pull down assay was implemented to testify the binding capacity between LINC00992 and miR-3935. **h** miR-3935 overexpression efficiency and inhibition efficiency were examined by qRT-PCR. **i** RIP assay disclosed the binding of miR-3935 to LINC00992 in the anti-Ago2 group. **j** The potential binding site between LINC00992 and miR-3935 was shown. And the luciferase activity of LINC00992-WT or LINC00992-MUT reporter was assessed via luciferase reporter assay in DU145 and PC3 cells after transfection with miR-3935-mimics, miR-3935-inhibitor, NC-inhibitor or NC-mimics. *P < 0.05, **p < 0.01, ***p < 0.001

Given the high correlation of the sub-localization of LINC00992 with its functional mechanism, the predication of LINC00992 presence in cells was performed via LncLocator (http://www.csbio.sjtu.edu.cn/bioinf/lncLocator/). Result predicted that LINC00992 located mainly in cytoplasm (Fig. 2a). Likewise, FISH assay and RNA isolation of nuclear and cytoplasmic fractions further verified the abundance of LINC00992 in the cytoplasm of prostate cancer cells (Fig. 2b-c), highlighting a post-transcriptional control of LINC00992 in such cells. Hence, we speculated that LINC00992 might act as a ceRNA in prostate cancer regulation. According to DIANA-lncBase, the top three potential miRNAs possessing the binding capacity with LINC00992 were listed (Fig. 2d). To target the highly-matched miRNA to LINC00992, qRT-PCR analysis was conducted to test the expression changes of these miRNAs following either LINC00992 depletion or augmentation. The results demonstrated that only miR-3935 expression was increased by LINC00992 depletion (Fig. 2e), but reduced by LINC00992 overexpression in the meantime (Fig. 2f). Thus, miR-3935 was chosen for further analysis. Afterwards, RNA pull down assay was implemented and the result depicted that LINC00992 was pulled down by Bio-miR-3935-WT (Fig. 2g), which indicated the binding of LINC00992 and miR-3935. Later, we observed the satisfactory efficiency of miR-3935 overexpression and miR-3935 inhibition through qRT-PCR analysis (Fig. 2h). Thereafter, RIP assay applying anti-Ago2 was executed. Results illustrated that LINC00992 and miR-3935 were highly enriched in anti-Ago2 group in comparison with control antibody (Fig. 2i), certifying the association of LINC00992 with miR-3935 in the RNA-induced silencing complexes (RISCs). To further explore the interaction between LINC00992 and miR-3935, the binding sites between LINC00992 and miR-3935 were predicted at first, and then data from luciferase reporter assay revealed that miR-3935 upregulation decreased the luciferase activity of LINC00992-WT reporter whereas miR-3935 inhibition increased the luciferase activity of LINC00992-WT reporter (Fig. 2j). Altogether, LINC00992 combined with miR-3935 to act as a miRNA decoy in prostate cancer.

### LINC00992 regulates the expression of GOLM1, a target of miR-3935

**Fig. 3:**
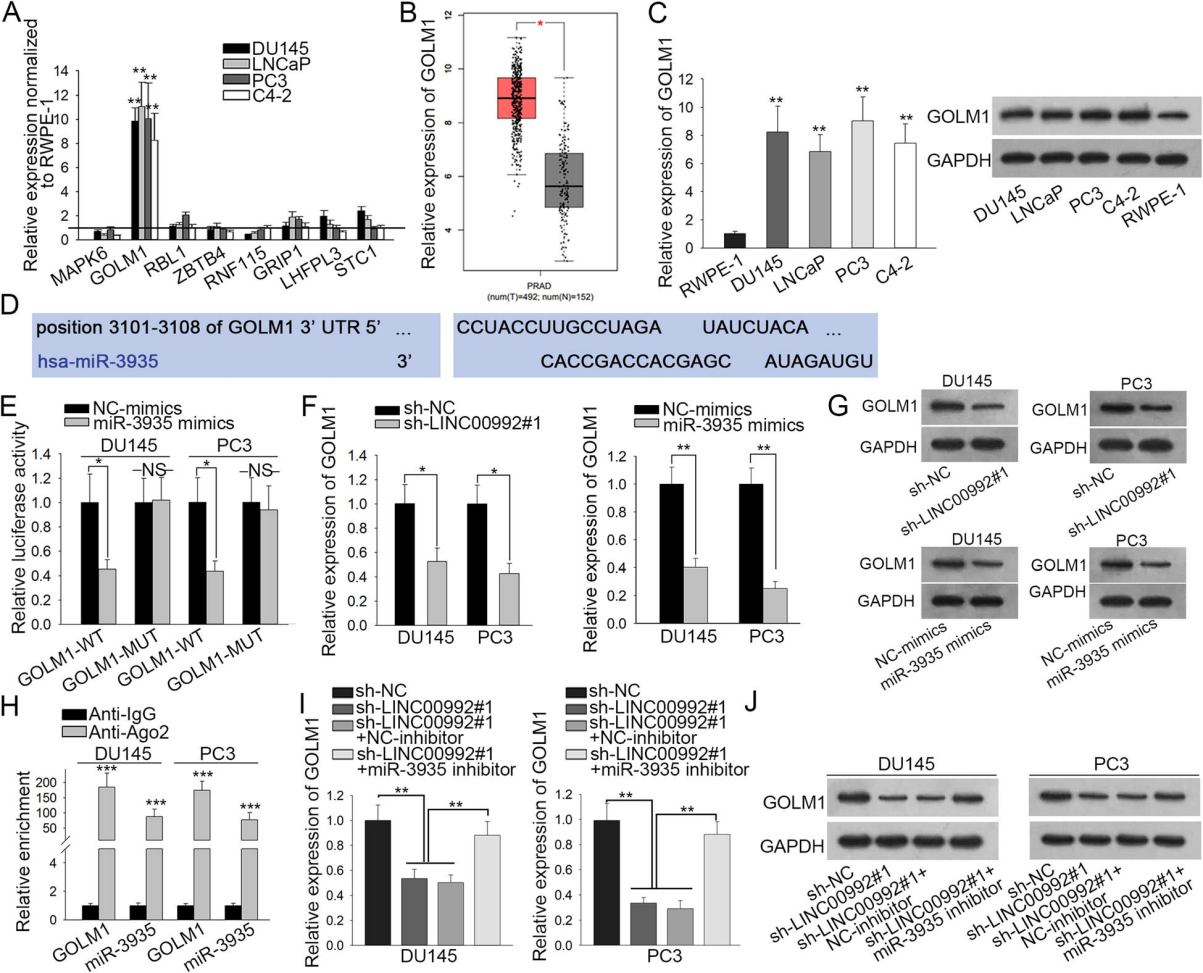
LINC00992 regulated the expression of GOLM1, a target of miR-3935. **a** The expression of eight mRNAs in four prostate cancer cell lines and RWPE-1 cells was detected by qRT-PCR. **b** GOLM1 was overexpressed in prostate cancer tissues according to GEPIA database. **c** The mRNA and protein levels of GOLM1 were evaluated in prostate cancer cell lines and RWPE-1 cell line by qRT-PCR and western blot, respectively. **d** The binding sites between GOLM1 and miR-3935 were predicted via TargetScan. **e** Luciferase reporter assay presented the inhibited luciferase activity of GOLM1-WT reporter in the presence of miR-3935 mimics not NC-mimics. **f-g** GOLM1 expression in transfected cells was tested by qRT-PCR and western blot analyses. **h** The combination of GOLM1 with miR-3935 in the anti-Ago2 group was validated by RIP assay. i-j The mRNA and protein levels of GOLM1 in different groups were examined via qRT-PCR and western blot. The full-length gels for western blot data in Fig. 3c, g and j were presented in Supplementary Figure 5. *P < 0.05, **p < 0.01, ***p < 0.001

Present evidence has suggested that miRNAs can bind with downstream target genes to inhibit their expression. Herein, we searched the miR-3935 target genes, and eight mRNAs were found out. Subsequently, we detected their expression in prostate cancer cells and normal cells. Interestingly, we found that only Golgi membrane protein 1 (GOLM1) was highly expressed in four prostate cancer cell lines relative to normal controls (Fig. 3a). Further, we discovered that GOLM1 expression was markedly upregulated in prostate cancer tissues according to data from GEPIA database (Fig. 3b). Similarly, GOLM1 expression was much higher in prostate cancer tissue samples than in peri-tumor samples (Figure S3A). In addition, the mRNA and protein levels of GOLM1 were overexpressed in prostate cancer cells in contrast to RWPE-1 cells (Fig. 3c, Figure S3B). Besides, GOLM1 has been previously revealed as a prostate cancer facilitator and was metastasis-related in prostate tumor [25–29]. Thus, we hypothesized that GOLM1 might act as the downstream of LINC00992/miR-3935 signaling in prostate cancer. Through TargetScan (http://www.targetscan.org/vert_72/), the binding site between GOLM1 and miR-3935 was predicted (Fig. 3d). After conducting luciferase reporter assay in DU145 and PC3 cells, we observed that upregulation of miR-3935 specifically decreased the luciferase activity of GOLM1-WT reporter (Fig. 3e), confirming the interaction between miR-3935 and GOLM1 relied on the putative binding sites. Then, we unveiled that GOLM1 mRNA and protein levels were both reduced by LINC00992 inhibition or miR-3935 upregulation according to qRT-PCR and western blot analyses (Fig. 3f-g, Figure S3C). Moreover, data from RIP assay unveiled the binding of miR-3935 to GOLM1 in the RISCs (Fig. 3h). Further, we demonstrated that the decreased mRNA and protein levels of GOLM1 induced by LINC00992 depletion could be restored after inhibiting miR-3935 expression (Fig. 3i-j, Figure S3D). All the results showed that LINC00992 up-regulated GOLM1 expression via directly binding to miR-3935.

### LINC00992 promotes prostate cancer cell proliferation and migration via elevating GOLM1 expression

**Fig. 4:**
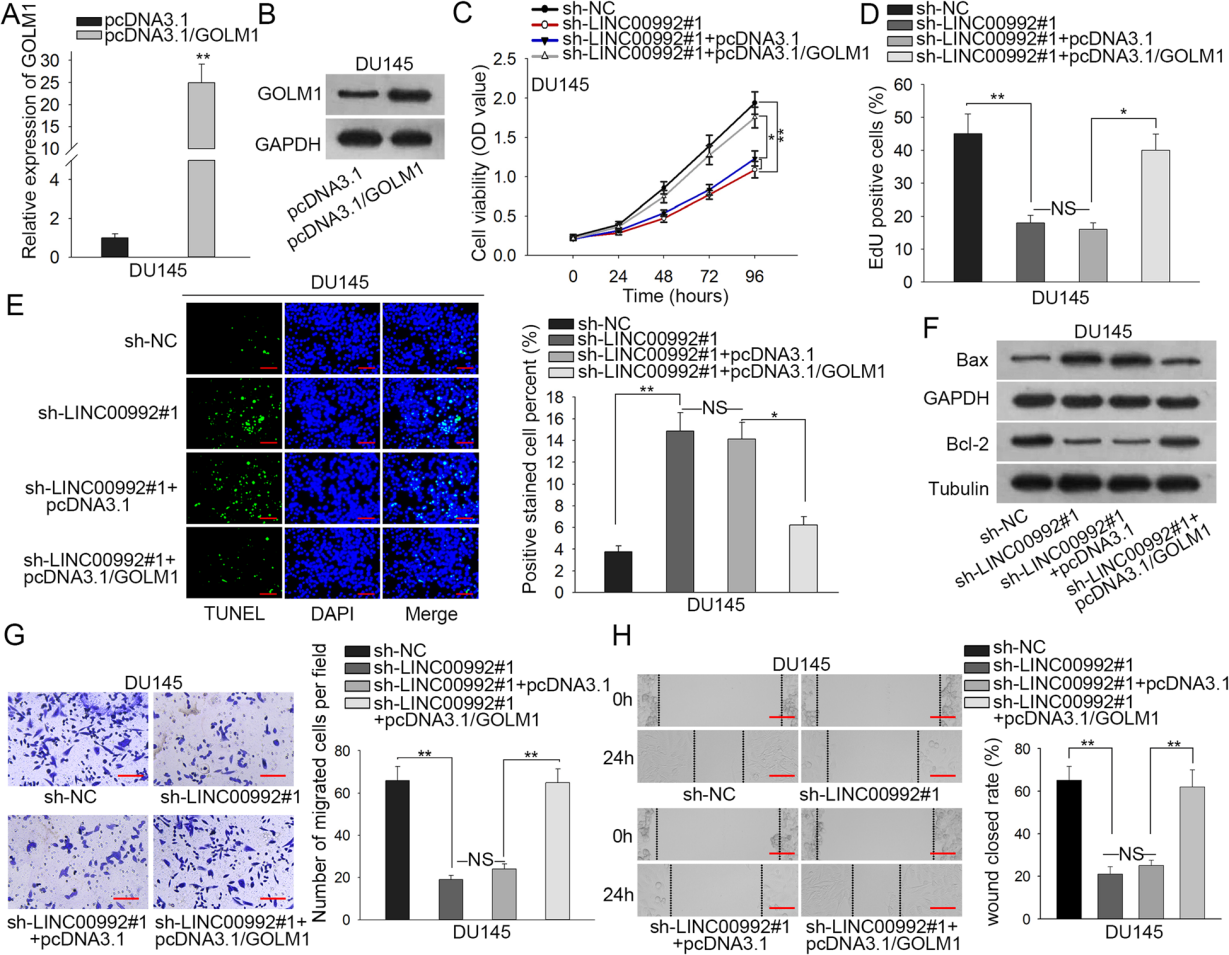
LINC00992 promoted prostate cancer cell proliferation and migration via elevating GOLM1 expression. **a-b** GOLM1 mRNA and protein levels in DU145 cells transfected with pcDNA3.1 or pcDNA3.1/GOLM1 were detected via qRT-PCR and western blot. pcDNA3.1 served as the negative control. **c** The viability of DU145 cells was determined via CCK-8 following transfection of different plasmids. **d** The proliferation of transfected cells was evaluated via EdU assay. **e** The apoptosis of transfected cells was monitored via TUNEL assay. Scale bar = 200 μm. **f** The protein levels of Bax and Bcl-2 in different groups were detected via western blot. **g-h** The migration of transfected cells was measured via Transwell migration assay (scale bar = 100 μm) and wound healing assay (scale bar = 100 μm). The full-length gels for western blot data in Fig. 4b and f were presented in Supplementary Figure 6. *P < 0.05, **p < 0.01

To test whether LINC00992 affected prostate cancer cell proliferation, apoptosis and migration via regulating miR-3935-targeted GOLM1, we executed the rescue experiments with the up-regulation of GOLM1. To begin with, the efficiency of overexpressing GOLM1 was analyzed through qRT-PCR and western blot analyses and the outcome turned out to be satisfactory (Fig. 4a-b, Figure S3E). Then we observed that overexpression of GOLM1 could significantly elevate the mRNA and protein expression of GOLM1 in sh-LINC00992#1-transfected cells (Figure S3F). Afterwards, data from CCK-8 revealed that the viability of DU145 cells was firstly hindered due to LINC00992 depletion while subsequent GOLM1 elevation reversed the inhibitory trend on DU145 cell viability (Fig. 4c). Results from EdU assay also exposed similar trends that GOLM1 up-regulation countervailed the suppressive impact posed by LINC00992 down-regulation on DU145 cell proliferation (Fig. 4d). Similarly, the restraining effect of silenced LINC00992 on the expression of proliferation-related proteins could be reversed by GOLM1 upregulation (Figure S3G). Later, TUNEL assay revealed that cell apoptosis rate was elevated by LINC00992 depletion and then overexpressing GOLM1 reduced the increased apoptosis rate of LINC00992-depleted cells (Fig. 4e). Likewise, western blot analysis uncovered that overexpressing GOLM1 could offset the effect of LINC00992 downregulation on the expression of apoptosis-related proteins (Fig. 4f, Figure S3H). Moreover, Transwell migration and wound healing assays illuminated that the retarding influence of silenced LINC00992 on cell migration could be rescued by GOLM1 overexpression (Fig. 4g-h). As expected, the inhibitory effect of LINC00992 depletion on the expression of migration-related molecular markers (MMP2, MMP9, p-Src and p-FAK) could be countervailed by GOLM1 overexpression (Figure S3I). Collectively, GOLM1 was required in LINC00992-regulated prostate cancer cellular processes.

### LINC00992 contributes to tumor growth via upregulating GOLM1 expression

**Fig. 5:**
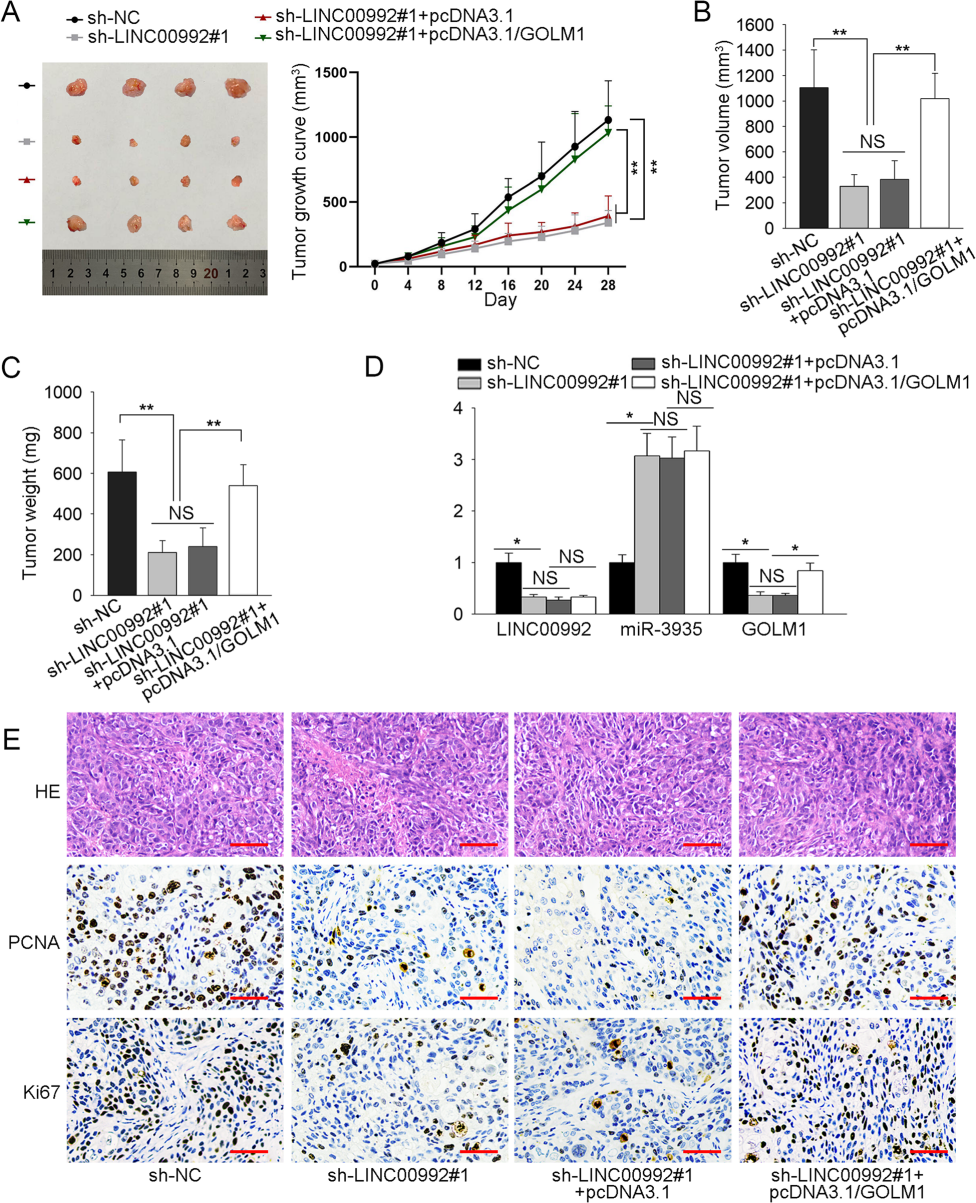
LINC00992 contributes to tumor growth via upregulating GOLM1 expression. **a** Representative images and the growth curves of tumors from indicated groups. **b-c** The volume and weight of tumors from above groups. **d** The expression of LINC00992, miR-3935 and GOLM1 in tumors from different groups was detected via qRT-PCR analysis. **e** The staining of PCNA and Ki67 in different groups was measured via IHC. Scale bar = 50 μm. **p < 0.01

After the in vitro exploration of LINC00992 performance in prostate cancer, we applied the in vivo assays to further validate above findings. As shown in Fig. 5a, tumors derived from LINC00992-silenced DU145 cells were smaller, with the growth rate quite slower, than those from control cells; more importantly, such blockage on tumor growth was obviously countervailed after GOLM1 overexpression. Besides, elevating GOLM1 expression could recover the lessened tumor volume and declined tumor weight induced by LINC00992 deficiency (Fig. 5b-c). Of note, we discovered decreased level of LINC00992 and enhanced level of miR-3935 in tumors from latter three groups compared to control group, while the lowered expression of GOLM1 in tumors with LINC00992 inhibition was normalized under GOLM1 overexpression (Fig. 5d). In addition, the inhibitory impact of silenced LINC00992 on the positivity of proliferation-associated proteins (PCNA and Ki67) could be reversed by upregulation of GOLM1 (Fig. 5e). Taken together, LINC00992 promoted the tumorigenesis of prostate cancer through upregulating GOLM1 expression.

## Discussion

As documented, the aberrant regulation of lncRNAs is a frequent event in diversified tumor types. Besides, the correlation between abnormal lncRNA expression and prostate cancer oncogenesis has also been extensively explored. For example, lncRNA SNHG7 facilitates prostate cancer carcinogenesis via cyclin D1 by sponging miR-503 [30]. LncRNA SChLAP1 aggravates prostate cancer cell proliferation and metastasis by targeting miR-198 [31]. LncRNA PCAT-1 contributes to prostate cancer tumorigenesis through modulating FSCN1 and sponging miR-145-5p [32]. In our work, LINC00992 was revealed to be highly expressed in prostate cancer tissues and cells, but unlike former investigations, our study gave a precise explanation about its role in prostate cancer. Our study unveiled that LINC00992 promoted cell proliferation and migration, whereas suppressed cell apoptosis in prostate cancer. Above-mentioned data validated that LINC00992 elicited a tumor-promoting function in prostate cancer.

Presently, accumulating evidence has indicated that cytoplasmic lncRNAs assisted the expression of downstream miRNA-targeted mRNAs via sponging the specific miRNAs. Before exploring LINC00992-mediated mechanism in prostate cancer, herein we firstly discovered its subcellular distribution in prostate cancer cells with both aids from online prediction tool (LncLocator) and experimental data (FISH and RNA isolation of nuclear and cytoplasmic fractions). Our study for the first time uncovered that LINC00992 located mainly in the cytoplasm of prostate cancer cells. Besides, our study also completed LINC00992-modulated mechanism by disclosing the downstream target miR-3935. The direct interaction between LINC00992 and miR-3935 was also first validated in this research.

In the subsequent search of mRNA targeted by miR-3935, we paid heed to Golgi Membrane Protein 1 (GOLM1). Previously, downregulation of GOLM1 inhibits glioblastoma cell proliferation and motility, and progression [33, 34]. GOLM1 aggravates cell growth and metastasis in breast cancer, and aggressiveness in non-small-cell carcinoma and hepatocellular carcinoma [35–37]. In lung adenocarcinoma, GOLM1 facilitates cell proliferation and represents unfavorable survival [38, 39]. In prostate cancer, GOLM1 has also been corroborated as tumor promoter [25–29]. Currently, we also revealed GOLM1 as upregulated in prostate cancer. Despite previously reported miRNAs targeting GOLM1 such as miR-382, miR-145, miR-200a, we first identified miR-3935 as the upstream regulator of GOLM1 in prostate tumor. Most importantly, rescue experiments highlighted that GOLM1 overexpression could rescue the suppressive effect of silenced LINC00992 on cell growth and migration in vitro and tumor growth in vivo.

## Conclusion

To sum up, all findings presented in the work contributed to a better grasp about the mechanism in prostate cancer carcinogenesis. It was elaborated that LINC00992 accelerated prostate cancer cell growth and migration via sponging miR-3935 and therefore promoting GOLM1 expression, providing new promising therapeutic biomarkers for prostate cancer. Nevertheless, current study lacks the evidence confirming the regulation of LINC00992 on tumor metastasis in vivo, which will motivate us to explore further in the future study.

## Supplementary information

**Additional file 1: Supplementary Figure 1.** (A) qRT-PCR analysis indicated the upregulation of LINC00992 in prostate cancer tissue samples in contrast to peri-tumor samples. (B) The association of LINC00992 expression with overall survival of prostate cancer patients was analyzed via Kaplan-Meier curve. (C) The expression of proliferation-related proteins in transfected cells was evaluated via western blot. (D) The quantification of immunoblots in Fig. 1g was shown. (E) The expression of migration-related molecular markers in transfected cells was analyzed via western blot. The full-length gels for western blot data in Figures S1C and S1E were presented in Supplementary Figure 7. **p < 0.01.

**Additional file 2: Supplementary Figure 2.** (A) The efficiency of LINC00992 overexpression was assessed via qRT-PCR in RWPE-1 cells. (B-C) The proliferation ability of transfected cells was estimated via CCK-8 and EdU assays (scale bar = 200 μm). (D) The expression of proliferation-related proteins in different groups was evaluated via western blot. (E-F) Transwell and wound healing assays were applied to analyze the migration ability of transfected cells. Scale bar = 100 μm. (G) The expression of migration-related molecular markers in different groups was analyzed via western blot. The full-length gels for western blot data in Figures S2D and S2G were presented in Supplementary Figure 8. **p < 0.01.

**Additional file 3: Supplementary Figure 3.** (A) qRT-PCR analysis indicated the upregulation of GOLM1 in prostate cancer tissue samples in contrast to peri-tumor samples. (B-E) The immunoblots in Figs. 3c, g, j and 4b was quantified. (F) The mRNA and protein levels of GOLM1 in different groups were detected via qRT-PCR and western blot. (G) The expression of proliferation-related proteins in different groups was evaluated via western blot. (H) The quantification of immunoblots in Fig. 4f was displayed. (I) The expression of migration-related molecular markers in different groups was analyzed via western blot. The full-length gels for western blot data in Figures S3F, S3G and S3I were presented in Supplementary Figure 9. **p < 0.01.

**Additional file 4: Supplementary Figure 4.** The full-length gel images of western blots in Fig. 1g.

**Additional file 5: Supplementary Figure 5.** The full-length gel images of western blot data in Fig. 3c, g and j.

**Additional file 6: Supplementary Figure 6.** The full-length images of western blots in Fig. 4b and f.

**Additional file 7: Supplementary Figure 7.** The full-length images of western blot data in Supplementary Figure 1C and E.

**Additional file 8: Supplementary Figure 8.** The full-length images of western blot data in Supplementary Figure 2D and G.

**Additional file 9: Supplementary Figure 9.** The full-length images of western blot data in Supplementary Figure 3F, G and I.

**Additional file 10: Supplementary file 1.** The enlarged images of picture data in Fig. 1e, f, h and i.

**Additional file 11: Supplementary file 2.** The enlarged images of picture data in Figs. 2b, 4e, g and h.

## Data Availability

All data generated or analysed during this study are included in this published article and its supplementary information files.

## Abbreviations

lncRNAs: Long non-coding RNAs
CCK-8: Cell Counting Kit-8
RIP: RNA immunoprecipitation
qRT-PCR: Quantitative real-time PCR
FISH: Fluorescence in situ hybridization assay
EdU: 5′-ethynyl-2′-deoxyuridine incorporation assay
TUNEL: Transferase-mediated dUTP nick end labeling staining
LINC00992: LncRNA long intergenic non-protein coding RNA 992
GOLM1: Golgi membrane protein 1
