# Supplementary figures and images for: LINC00992 contributes to the oncogenic phenotypes in prostate cancer via targeting miR-3935 and augmenting GOLM1 expression
Source: BMC Cancer. 2020 Aug 11;20:749. doi: 10.1186/s12885-020-07141-4 (PMC7418399; doi:10.1186/s12885-020-07141-4)

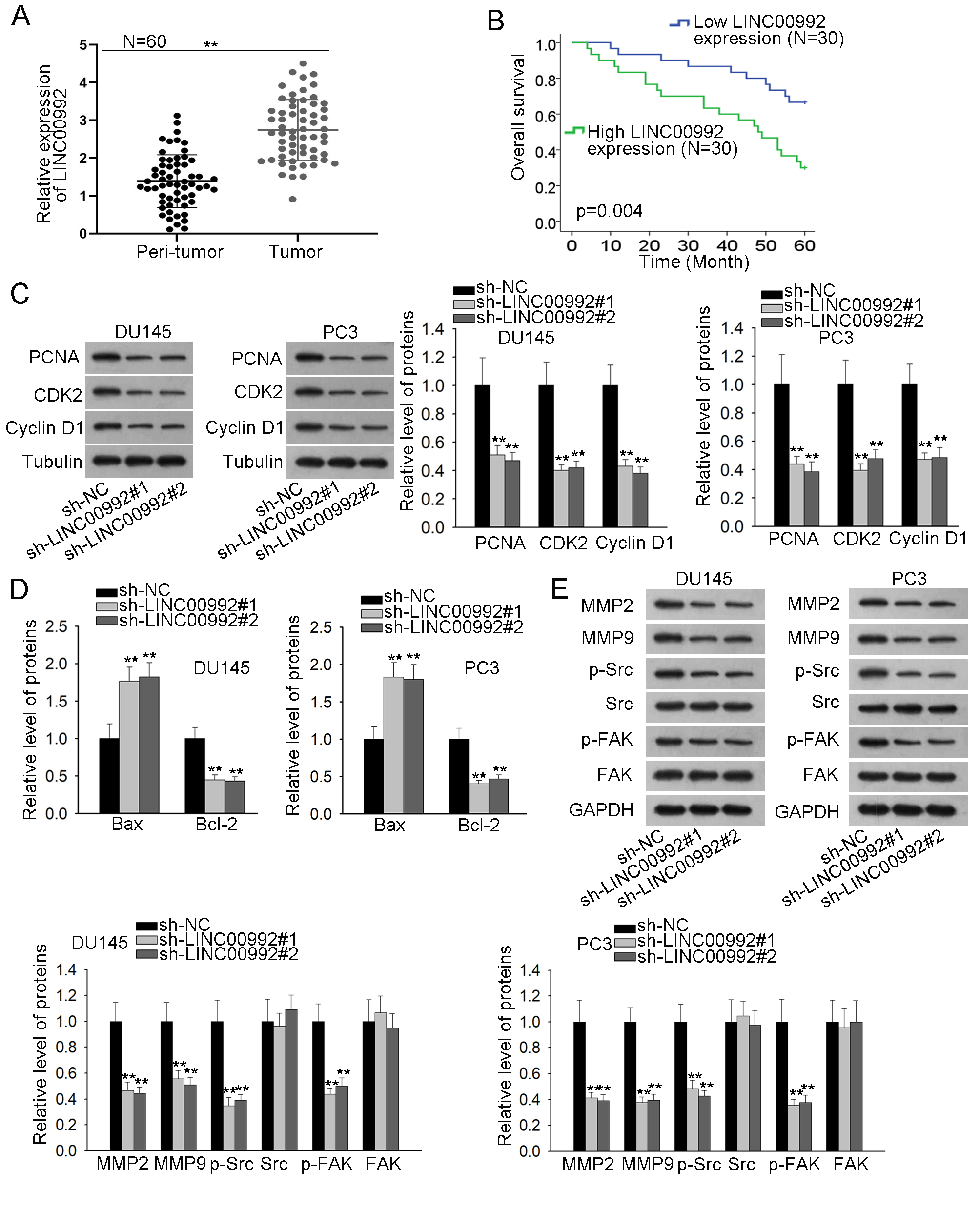

Supplement: Supplementary file 1 — Additional file 1: Supplementary Figure 1. (A) qRT-PCR analysis indicated the upregulation of LINC00992 in prostate cancer tissue samples in contrast to peri-tumor samples. (B) The association of LINC00992 expression with overall survival of prostate cancer patients was analyzed via Kaplan-Meier curve. (C) The expression of proliferation-related proteins in transfected cells was evaluated via western blot. (D) The quantification of immunoblots in Fig. 1g was shown. (E) The expression of migration-related molecular markers in transfected cells was analyzed via western blot. The full-length gels for western blot data in Figures S1C and S1E were presented in Supplementary Figure 7. **p < 0.01. [file 12885_2020_7141_MOESM1_ESM.tif]

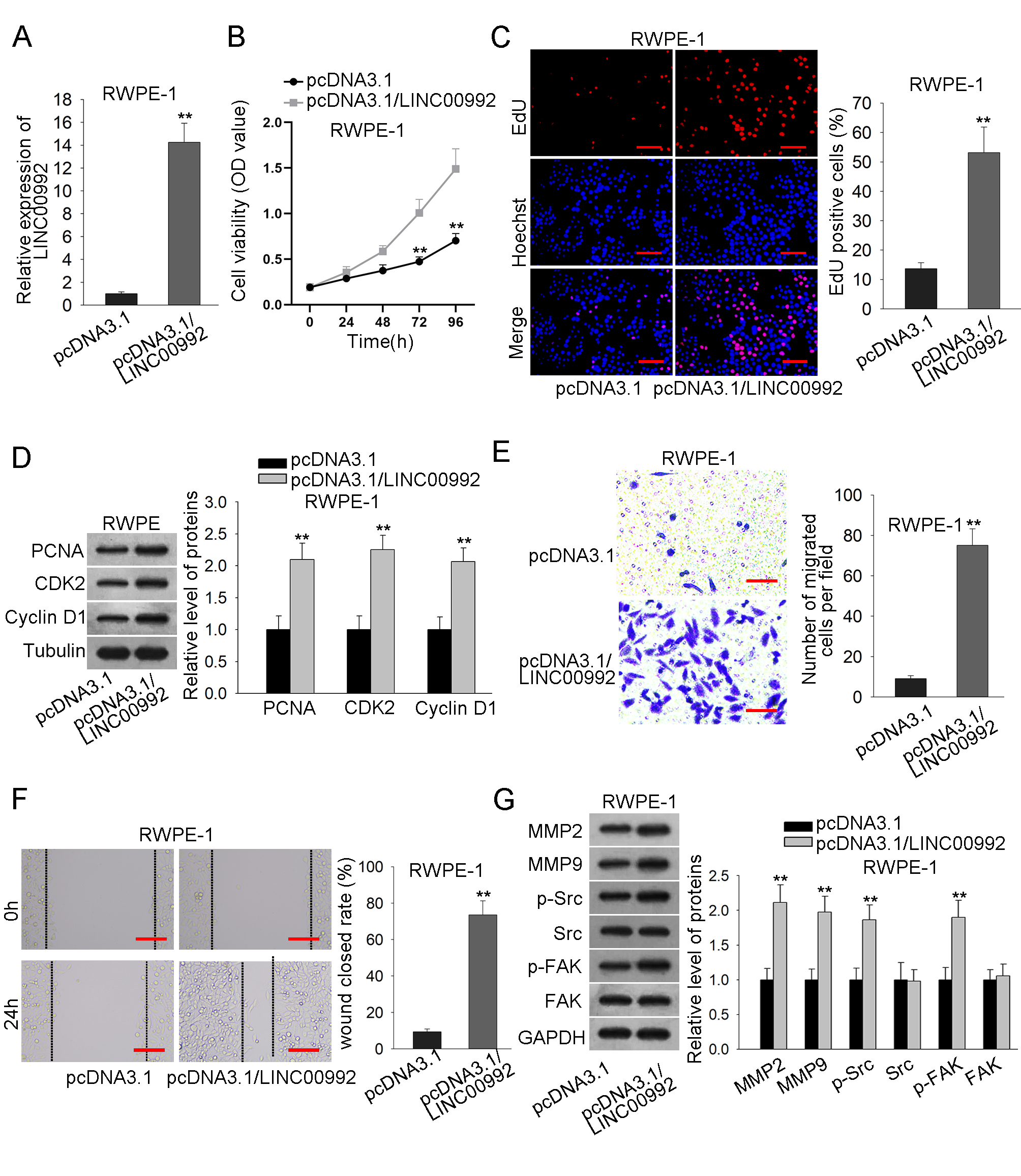

Supplement: Supplementary file 2 — Additional file 2: Supplementary Figure 2. (A) The efficiency of LINC00992 overexpression was assessed via qRT-PCR in RWPE-1 cells. (B-C) The proliferation ability of transfected cells was estimated via CCK-8 and EdU assays (scale bar = 200 μm). (D) The expression of proliferation-related proteins in different groups was evaluated via western blot. (E-F) Transwell and wound healing assays were applied to analyze the migration ability of transfected cells. Scale bar = 100 μm. (G) The expression of migration-related molecular markers in different groups was analyzed via western blot. The full-length gels for western blot data in Figures S2D and S2G were presented in Supplementary Figure 8. **p < 0.01. [file 12885_2020_7141_MOESM2_ESM.tif]

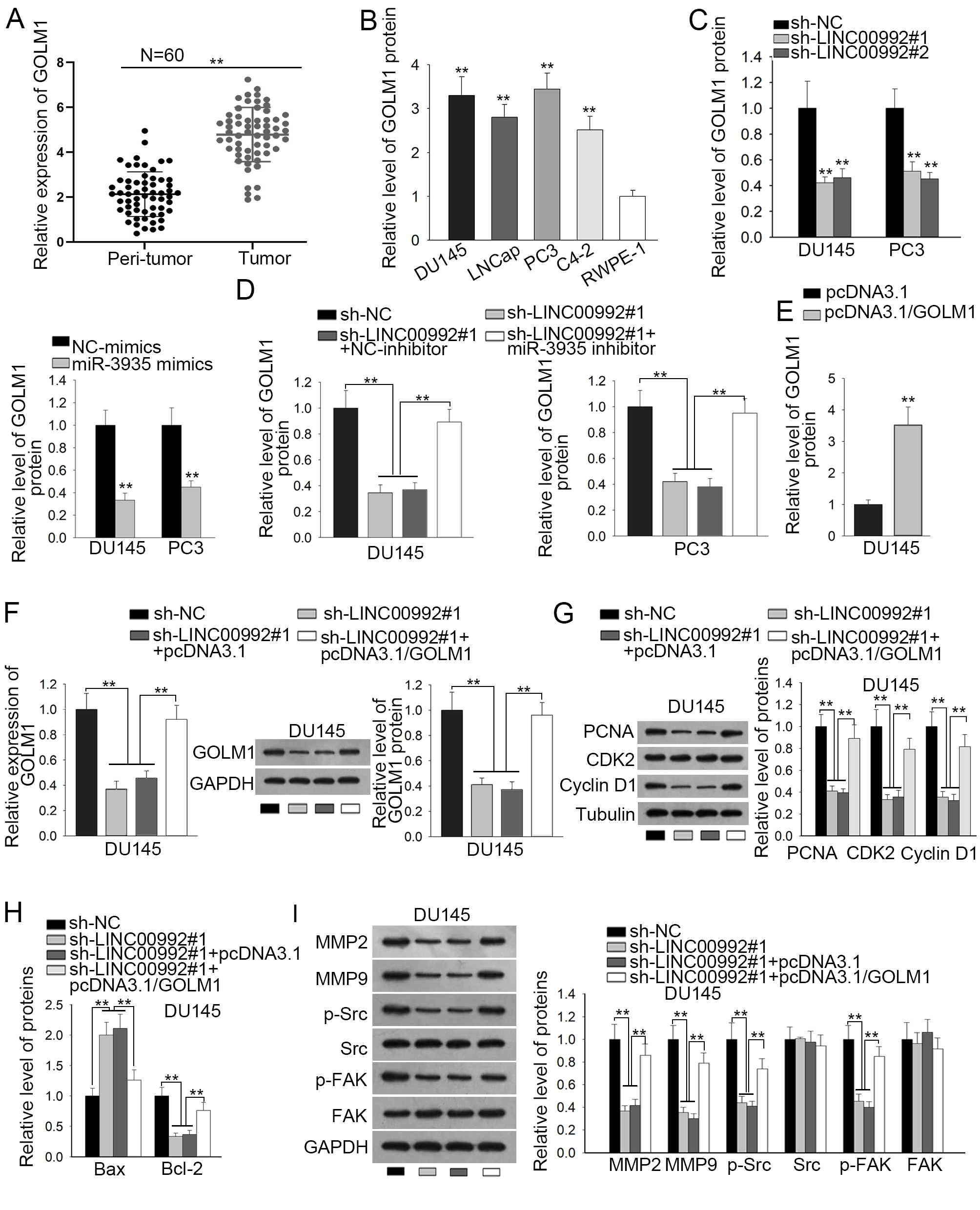

Supplement: Supplementary file 3 — Additional file 3: Supplementary Figure 3. (A) qRT-PCR analysis indicated the upregulation of GOLM1 in prostate cancer tissue samples in contrast to peri-tumor samples. (B-E) The immunoblots in Figs. 3c, g, j and 4b was quantified. (F) The mRNA and protein levels of GOLM1 in different groups were detected via qRT-PCR and western blot. (G) The expression of proliferation-related proteins in different groups was evaluated via western blot. (H) The quantification of immunoblots in Fig. 4f was displayed. (I) The expression of migration-related molecular markers in different groups was analyzed via western blot. The full-length gels for western blot data in Figures S3F, S3G and S3I were presented in Supplementary Figure 9. **p < 0.01. [file 12885_2020_7141_MOESM3_ESM.tif]

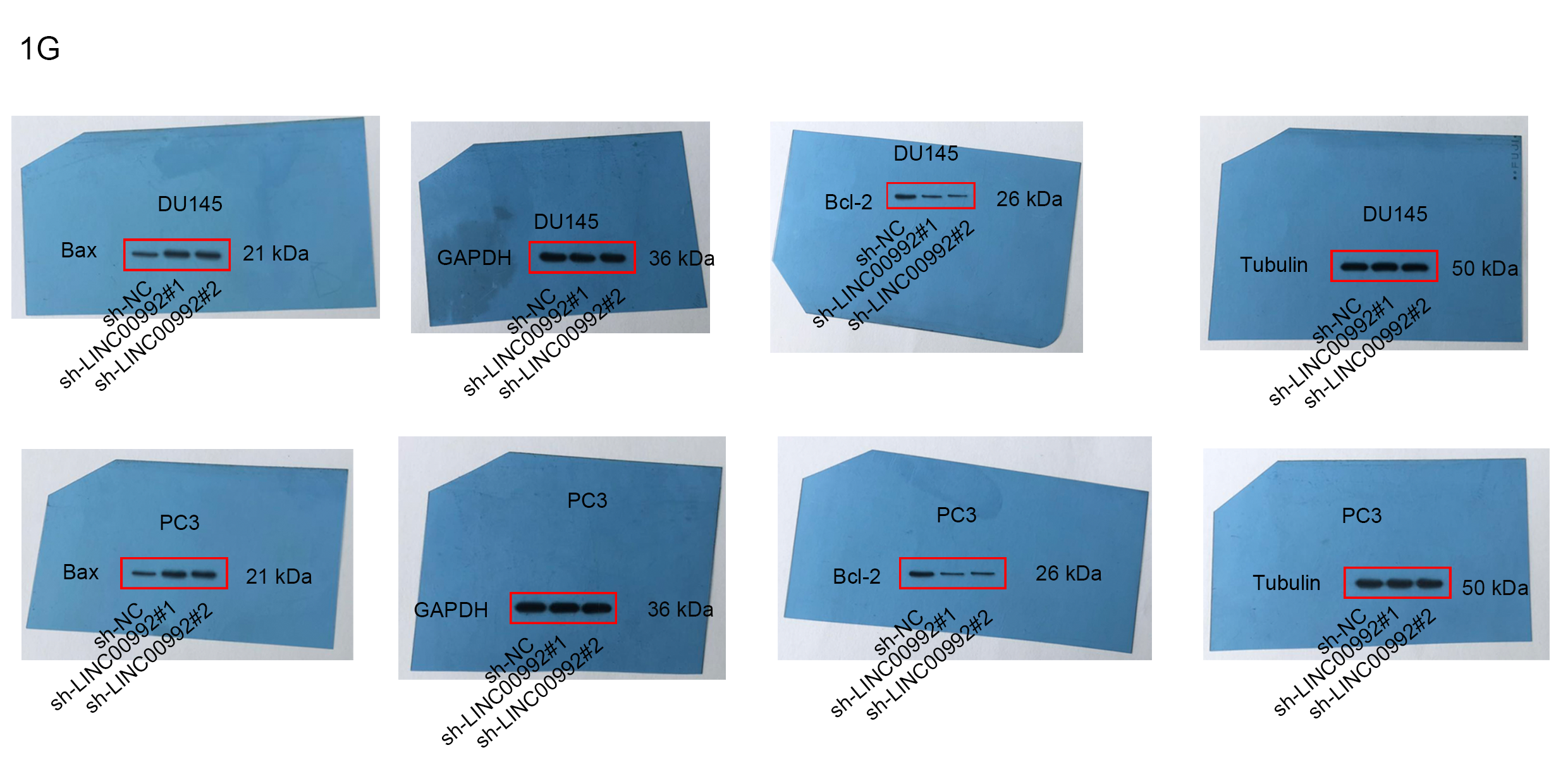

Supplement: Supplementary file 4 — Additional file 4: Supplementary Figure 4. The full-length gel images of western blots in Fig. 1g. [file 12885_2020_7141_MOESM4_ESM.tif]

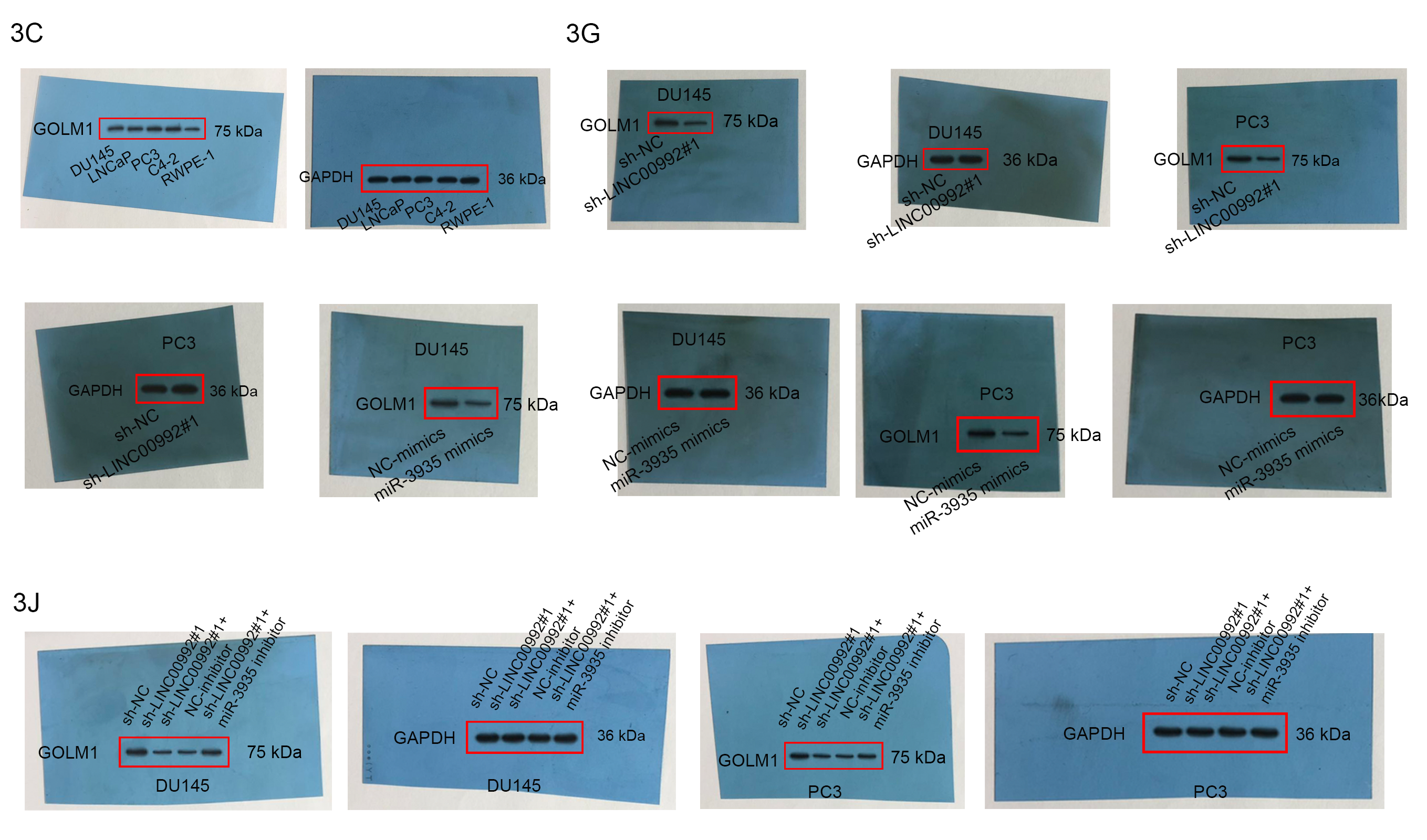

Supplement: Supplementary file 5 — Additional file 5: Supplementary Figure 5. The full-length gel images of western blot data in Fig. 3c, g and j. [file 12885_2020_7141_MOESM5_ESM.tif]

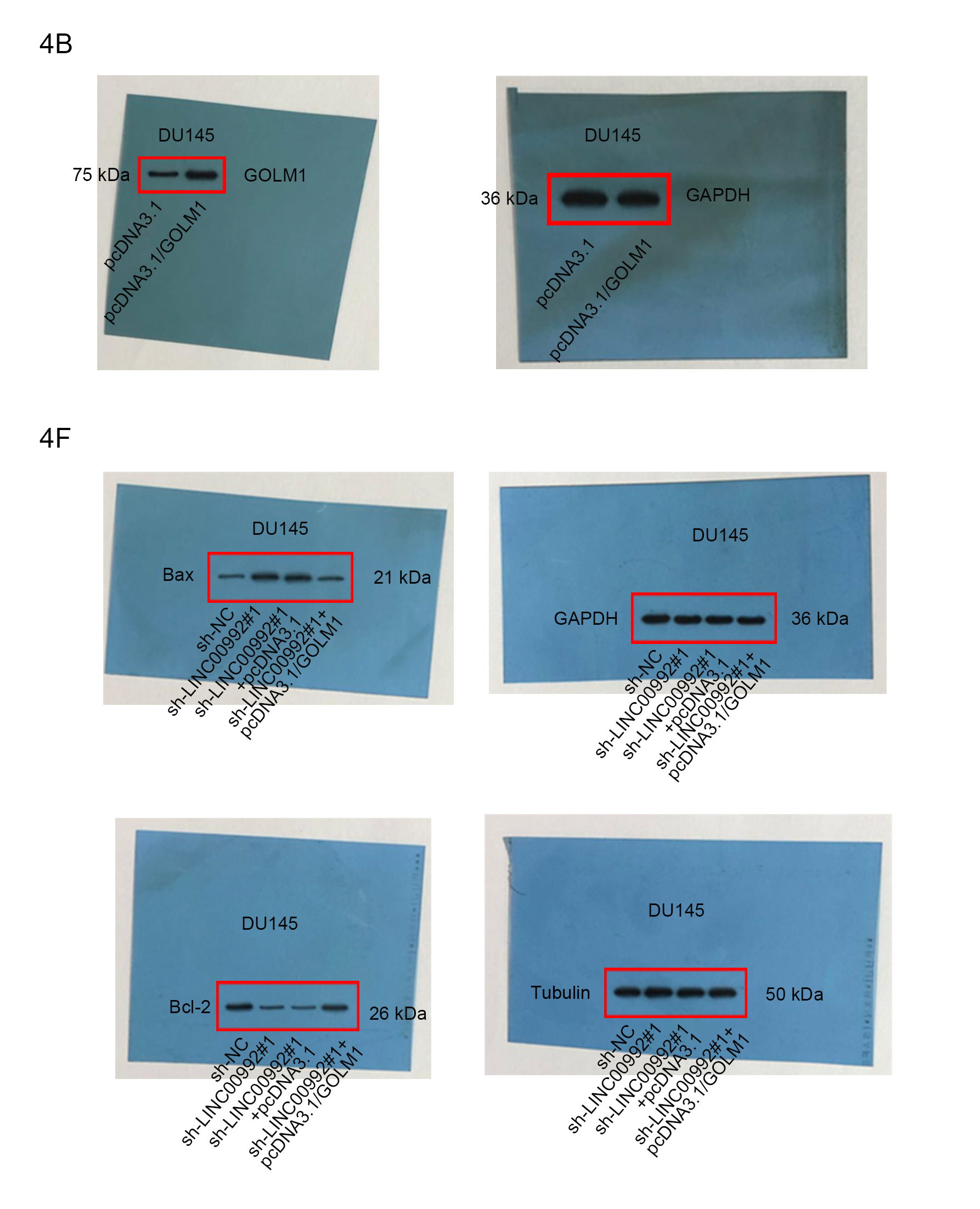

Supplement: Supplementary file 6 — Additional file 6: Supplementary Figure 6. The full-length images of western blots in Fig. 4b and f. [file 12885_2020_7141_MOESM6_ESM.tif]

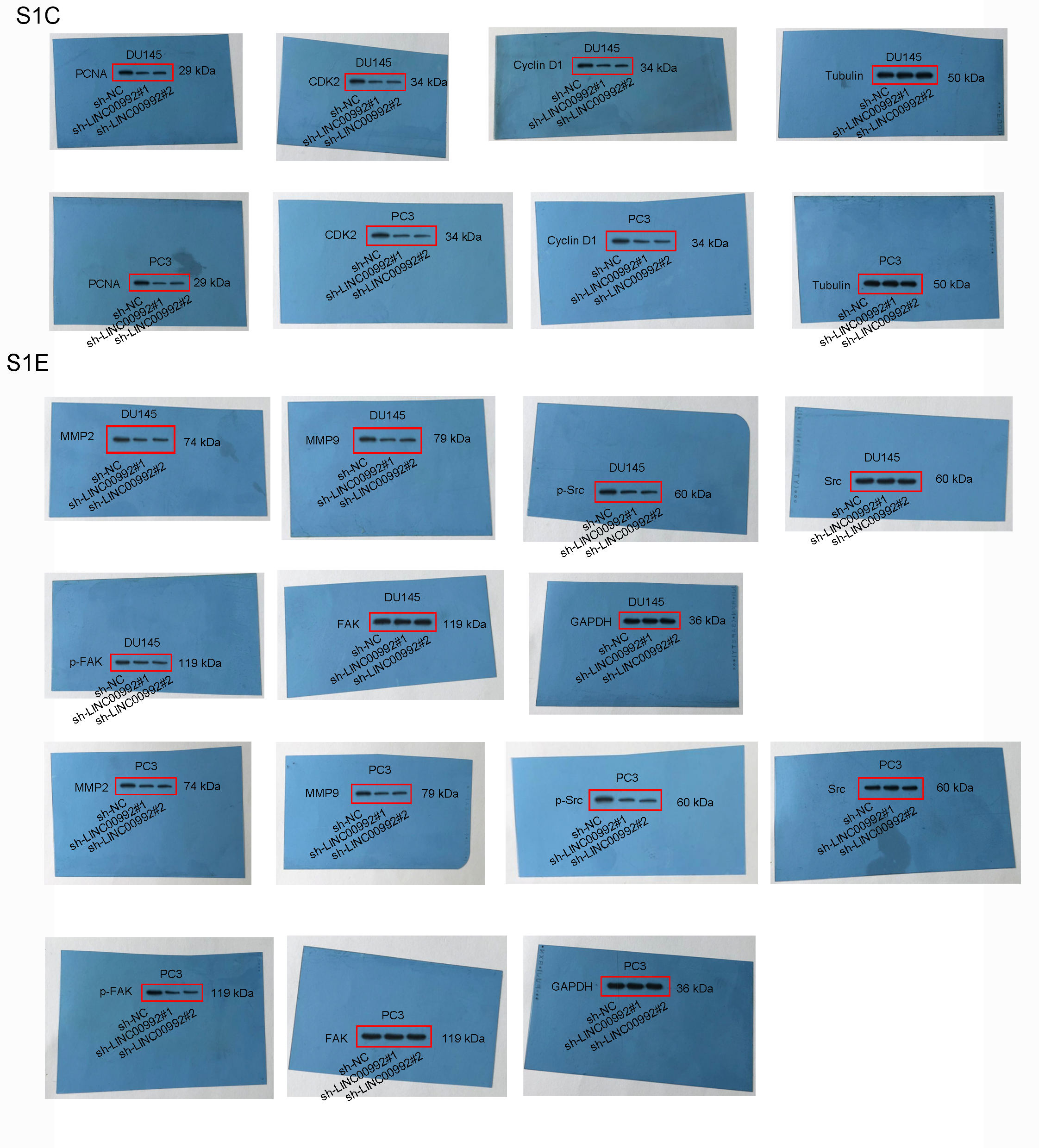

Supplement: Supplementary file 7 — Additional file 7: Supplementary Figure 7. The full-length images of western blot data in Supplementary Figure 1C and E. [file 12885_2020_7141_MOESM7_ESM.tif]

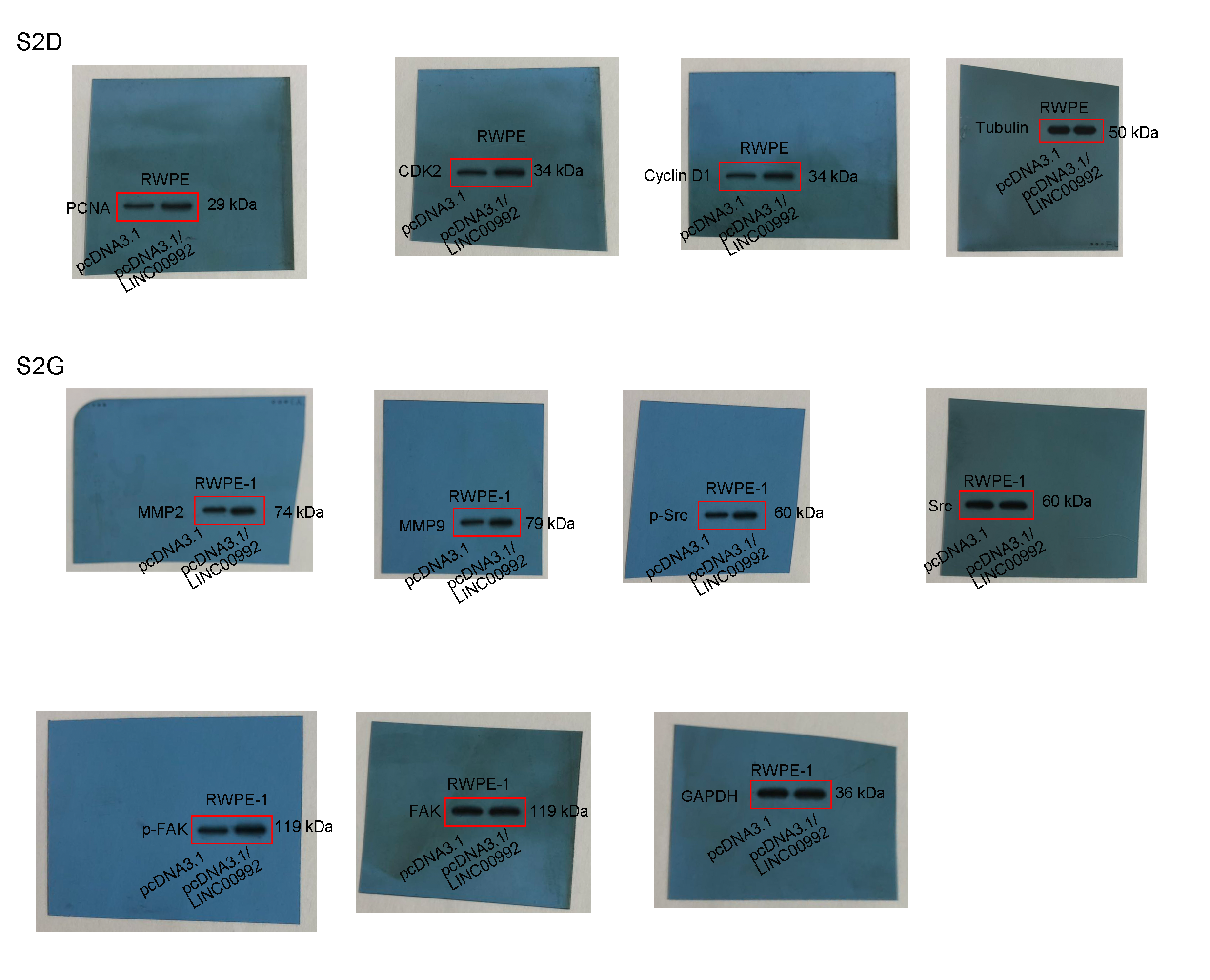

Supplement: Supplementary file 8 — Additional file 8: Supplementary Figure 8. The full-length images of western blot data in Supplementary Figure 2D and G. [file 12885_2020_7141_MOESM8_ESM.tif]

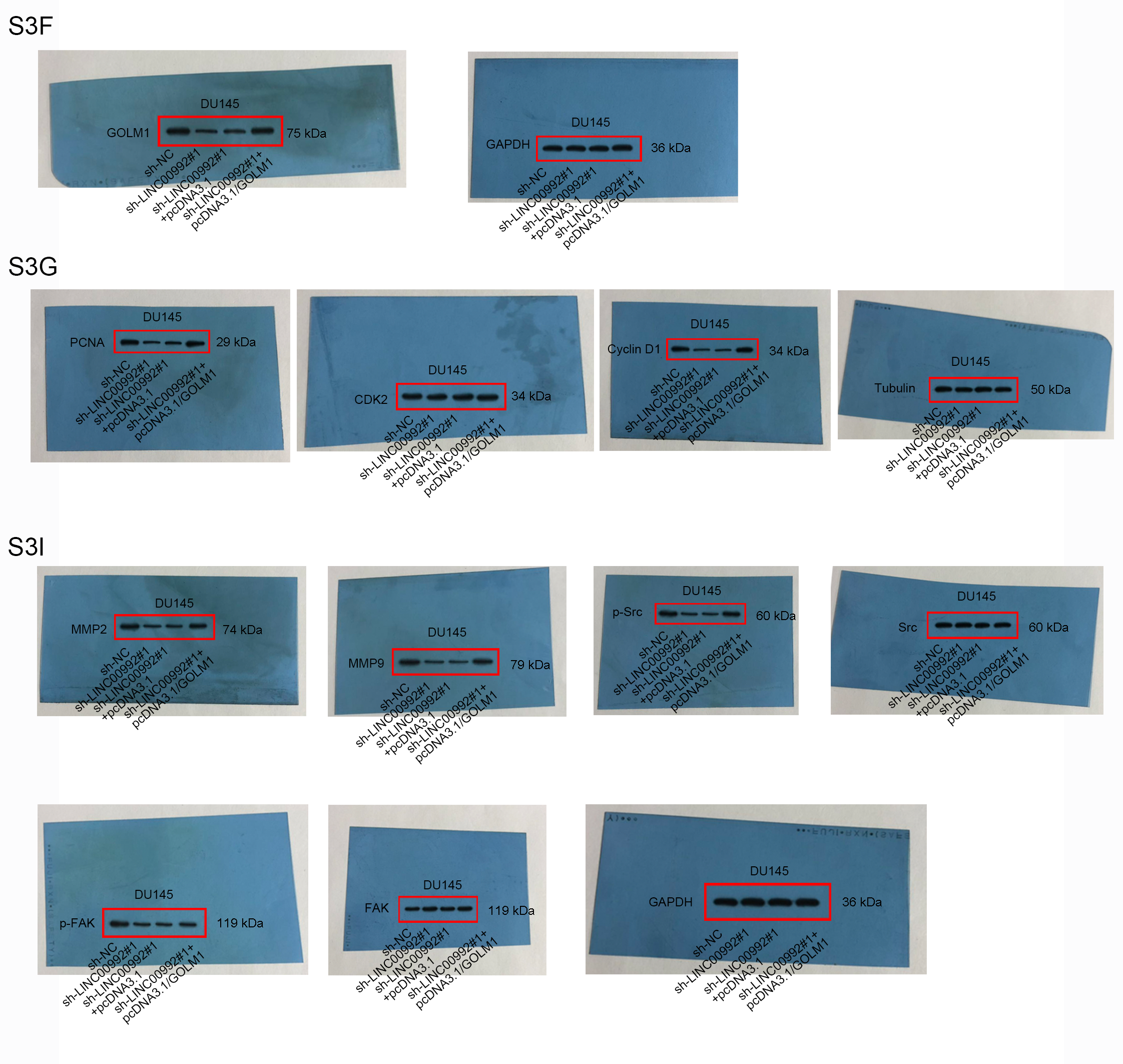

Supplement: Supplementary file 9 — Additional file 9: Supplementary Figure 9. The full-length images of western blot data in Supplementary Figure 3F, G and I. [file 12885_2020_7141_MOESM9_ESM.tif]

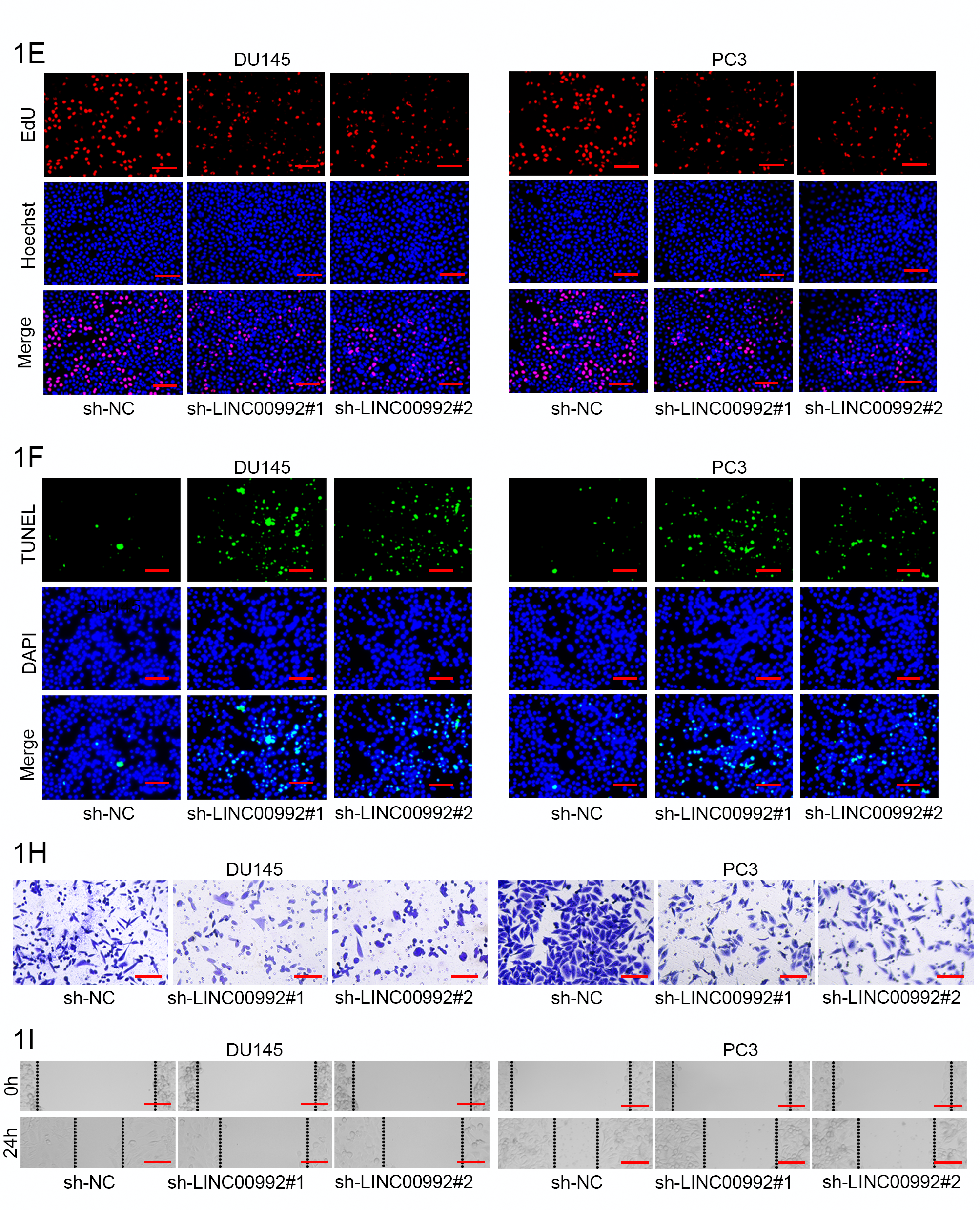

Supplement: Supplementary file 10 — Additional file 10: Supplementary file 1. The enlarged images of picture data in Fig. 1e, f, h and i. [file 12885_2020_7141_MOESM10_ESM.tif]

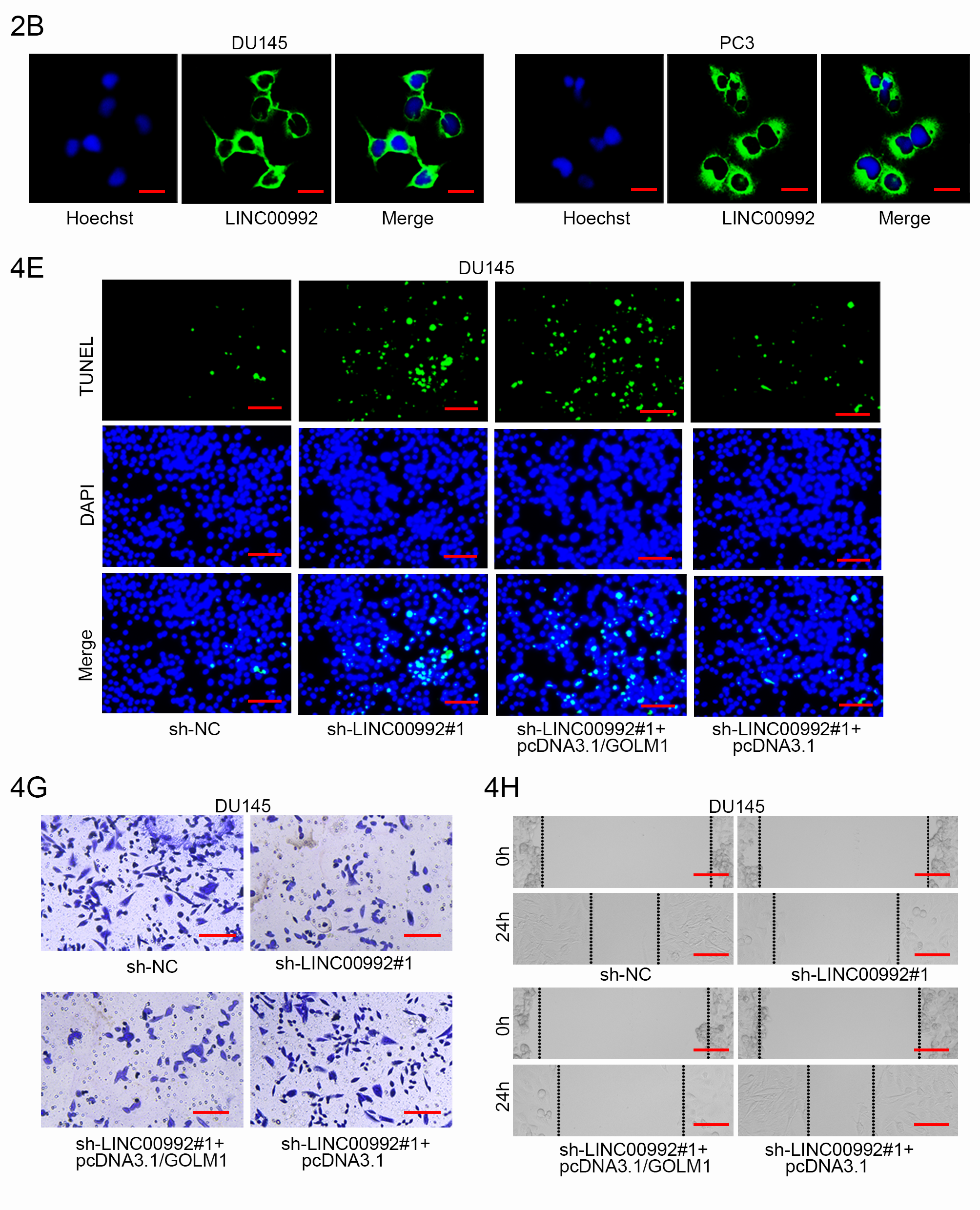

Supplement: Supplementary file 11 — Additional file 11: Supplementary file 2. The enlarged images of picture data in Figs. 2b, 4e, g and h. [file 12885_2020_7141_MOESM11_ESM.tif]
